# Endocannabinoid 2-Arachidonoylglycerol Self-Administration by Sprague-Dawley Rats and Stimulation of *in vivo* Dopamine Transmission in the Nucleus Accumbens Shell

**DOI:** 10.3389/fpsyt.2014.00140

**Published:** 2014-10-17

**Authors:** Maria Antonietta De Luca, Valentina Valentini, Zisis Bimpisidis, Fabio Cacciapaglia, Pierluigi Caboni, Gaetano Di Chiara

**Affiliations:** ^1^Neuropsychopharmacology Section, Department of Biomedical Sciences, University of Cagliari, Cagliari, Italy; ^2^National Institute of Neuroscience (INN), Cagliari, Italy; ^3^Centre of Excellence for Studies on the Neurobiology of Addiction, Cagliari, Italy; ^4^Department of Life and Environmental Sciences, University of Cagliari, Cagliari, Italy; ^5^Cagliari Section, Neuroscience Institute, National Research Council of Italy, Cagliari, Italy

**Keywords:** endocannabinoids, 2-arachidonoilglycerol, self-administration, reward, drug addiction, *in vivo* microdialysis, nucleus accumbens

## Abstract

2-Arachidonoylglycerol (2-AG) is the most potent endogenous ligand of brain cannabinoid CB_1_ receptors and is synthesized on demand from 2-arachidonate-containing phosphoinositides by the action of diacylglycerol lipase in response to increased intracellular calcium. Several studies indicate that the endocannabinoid (eCB) system is involved in the mechanism of reward and that diverse drugs of abuse increase brain eCB levels. In addition, eCB are self-administered (SA) by squirrel monkeys, and anandamide increases nucleus accumbens (NAc) shell dopamine (DA) in rats. To date, there is no evidence on the reinforcing effects of 2-AG and its effects on DA transmission in rodents. In order to fill this gap, we studied intravenous 2-AG SA and monitored the effect of 2-AG on extracellular DA in the NAc shell and core via microdialysis in male Sprague-Dawley rats. Rats were implanted with jugular catheters and trained to self-administer 2-AG [25 mg/kg/inf intravenously (iv)] in single daily 1 h sessions for 5 weeks under initial fixed ratio (FR) 1 schedule. The ratio was subsequently increased to FR2. Active nose poking increased from the 6th SA session (acquisition phase) but no significant increase of nose pokes was observed after FR2. When 2-AG was substituted for vehicle (25th SA session, extinction phase), rate responding as well as number of injections slowly decreased. When vehicle was replaced with 2-AG, SA behavior immediately recovered (reacquisition phase). The reinforcing effects of 2-AG in SA behavior were fully blocked by the CB1 receptor inverse agonist/antagonist rimonabant (1 mg/kg intraperitoneally, 30 min before SA session). In the microdialysis studies, we observed that 2-AG (0.1–1.0 mg/kg iv) preferentially stimulates NAc shell as compared to the NAc core. NAc shell DA increased by about 25% over basal value at the highest doses tested (0.5 and 1.0 mg/kg iv). The results obtained suggest that the eCB system, via 2-AG, plays an important role in reward.

## Introduction

Endocannabinoid (eCB) signaling controls various central functions in mammals, such as nociception, feeding, energy homeostasis, mood, learning, memory, growth, development, and reward processes (1–6). The eCB system consists of cannabinoid receptors (CB1 and CB2), lipid-derived endogenous ligands [*N*-arachidonoylethanolamine or anandamide, 2-arachidonoylglycerol (2-AG)], and specific enzymes involved in the biosynthesis and degradation of eCB.

Many questions on eCB signaling still need to be answered. However, among the two endogenous ligands, anandamide and 2-AG, 2-AG appears to be the most potent endogenous ligand for brain cannabinoid CB_1_ receptors (7). 2-AG acts as a full agonist of CB1 and CB2 receptors (8–10), and is synthesized on demand from 2-arachidonate-containing phosphoinositides by the action of diacylglycerol lipase (DAGLα and DAGLβ) in response to elevations of intracellular calcium. Unlike classic neurotransmission systems (e.g., monoaminergic, cholinergic, aminoacidergic), 2-AG signaling in the nervous system takes place in a retrograde fashion. Thus, stimulation of the postsynaptic neuron induces the biosynthesis of 2-AG that thus diffuses to the presynaptic terminal to act on CB1 receptors; due to its lipophilic nature, no synaptic vesicles for storage of 2-AG exist (4, 11, 12). Given these characteristics, 2-AG is considered to be a neuromodulator rather than a transmitter. CB1 activation of G_i/o_ proteins inhibits neurotransmission but the net inhibitory or excitatory effect of 2-AG signaling depends on the type of neurons involved in the process (4, 13–16). 2-AG is mainly degraded by monoacylglycerol lipase (MAGL), a membrane-associated, cytoplasm-facing soluble enzyme located pre-synaptically at axon terminals along with the CB1 receptor (17–19). Furthermore, about 15% of 2-AG is hydrolyzed by ABHD6 and ABHD12, and the remaining 1–2% by fatty acid amide hydrolase (FAAH) (20). The mechanisms of 2-AG neuronal reuptake are not completely known, but putative transporters have been described (3, 21).

Drugs of abuse affect brain eCB levels and it has been proposed that the activation of the eCB system is involved in many aspects of addiction (22). Drug-induced increases in eCB synthesis play a role in drug reward, and it has been suggested that eCBs are involved in long-term synaptic plasticity of neural substrates of motivation and reward in relation to addiction (23).

In human beings, a genetic disruption of eCB clearance is associated with drug abuse (24, 25). CB1 receptor activation affects the addictive properties of opioids, cocaine, alcohol, and nicotine (26). On the other hand, CB_1_ KO mice show reduced ethanol and morphine self-administration (SA) and attenuated ethanol- and opiate-induced place conditioning (27–29). In rats, the CB_1_ receptor inverse agonist/antagonist SR-141716A (Rimonabant) reduces ethanol and opiate SA (30–32) while in mice it reverses the behavioral and neurochemical effects of cocaine (33).

Δ9-Tetrahydrocannabinol (THC), the main active component of marijuana, is known to reduce the severity of opioid withdrawal in human beings and rodents, and acute inhibition of FAAH or MAGL alleviates symptoms of precipitated and spontaneous withdrawal in opioid- and THC-dependent mice (34, 35). The eCB system is involved in the rewarding effects of THC (22), as well as ethanol and opiates (36, 37). In particular, ethanol and heroin SA increases eCB levels in the nucleus accumbens (NAc) shell, suggesting a role for NAc eCB transmission in the reinforcing effects produced by these drugs (38). Finally, eCBs are SA by squirrel monkeys, meaning that they are effective reinforcers of drug-taking behavior (39, 40).

Dopamine (DA) neurotransmission is known to regulate a large number of motivated and addiction-related behaviors (41–43). Importantly, drugs of abuse of the most diverse pharmacological classes increase mesolimbic DA transmission in rats as well as in human beings preferentially or, depending on doses and conditions, selectively in the ventral striatum/NAc (42, 44–48). Accordingly, THC and the synthetic cannabinoid WIN 55,212-2 increase extracellular DA concentrations in the shell, but not in the core of the NAc, both when injected intravenously (iv) (49) and when SA by different strains of rats (50, 51). In addition, anandamide increases NAc shell DA and its effect is amplified by the FAAH inhibitor, URB597 (52). On the other hand, reduced DA transmission in the mesolimbic system is associated with spontaneous or rimonabant-precipitated THC withdrawal (53, 54).

In spite of this extensive literature, no studies are available on the reinforcing effects of 2-AG and its effects on DA transmission in rodents. In order to fill this gap, we studied intravenous 2-AG SA and monitored the effect of 2-AG on extracellular DA in the NAc shell and core via microdialysis in male Sprague-Dawley rats.

## Materials and Methods

### Animals

Male Sprague-Dawley rats (Harlan, Italy), weighting 250–275 g upon arrival, were housed four per cage given *ad libitum* access to food and water in a temperature (22°C) and humidity (60%) controlled vivarium with a 12 h light/dark cycle (on 08:00 A.M., off 08:00 P.M.). After surgery (catheter implantation), rats were individually housed in plastic cages (30 cm × 20 cm × 20 cm) given *ad libitum* food and water access, and in the same environmental conditions. For 7–10 days before surgery, rats were handled twice a day. SA sessions were performed during the light phase, between 9:00 a.m. and 5:00 p.m. After the experimental sessions, the rats were returned to their home cages where a daily ration of 18 g of food was made available, which maintained body weights at stable levels throughout these studies. The weight of rats at the beginning of SA studies was 300–325 g. Rats were weighed every day for the duration of the SA experiments. No significant reduction of body weight was observed. All experimental procedures met the guidelines and protocols approved by Italian (D.L. 116/92 and 152/06) and European Council directives (609/86 and 63/2010) and in compliance with the approved animal policies by the Ethical Committee for Animal Experiments (CESA, University of Cagliari) and the Italian Ministry of Health.

### Drugs

The eCB 2-AG was purchased from Tocris Cookson Ltd. (Northpoint, UK) and was dissolved in a vehicle containing 2% ethanol, 2% Tween 80, and saline and administered as an intravenous bolus of 20 μl for SA studies (12.5, 20, 50 μg/kg/infusion) or 1 ml/kg solution for microdialysis studies (0.1–1 mg/kg iv).

The CB1 receptor inverse agonist/antagonist rimonabant (SR-141716A) was obtained from Sigma (RD-Sigma, Italy) and suspended in 0.3% Tween 80 and saline. It was administered (1 mg/kg intraperitoneally, ip) 30 min prior to 2-AG SA sessions.

### 2-AG solutions

2-Arachidonoylglycerol content in the solutions prepared for SA or microdialysis studies was determined by HPLC–MS/MS analysis performed on MAX-RP C18 column (150 × 4.60 mm; 4 μm). The samples (20 μL) were analyzed by ESI in positive SIM mode following the ion [M + H]^+^ 379 *m*/*z*. The HPLC conditions were as follows: flow rate: 0.4 ml/min; solvent A: 0.1% formic acid in water; solvent B: acetonitrile; and gradient: solvent B 5–100% over 10 min. The samples (20 μl) were then analyzed by a Varian 1200 triple quadrupole HPLC–MS. Mass spectral data were acquired with a scan time of 1.0 s, needle 3500 V, shield 600 V, capillary 30 V, and detector 1900 V. The source parameters were adjusted as follows: drying gas temperature 250°C, drying gas pressure 20 psi, and nebulizer pressure 45 psi.

### Self-administration studies

Daily SA sessions were carried out in chambers housed in soundproof boxes (Coulbourn Instruments, Allentown, NJ, USA) containing two nose-poke holes, one active and the other inactive. A yellow/green light was placed over the active hole and a red light over the inactive one as discriminative stimuli. Prior to each daily session, the jugular catheter was flushed with 0.1 ml of sterile saline and the rats were placed in the SA box.

Rats were anesthetized with Equitesin (3 ml/kg ip; chloral hydrate 2.1 g, sodium pentobarbital 0.46 g, MgSO_4_ 1.06 g, propylene glycol 21.4 ml, ethanol (90%) 5.7 ml, H_2_O 3 ml) and implanted in the right jugular vein with a catheter, consisting of medical-grade tubing (Silastic, Dow Corning Corporation, Michigan, USA) according to the technique previously described (50). A stable fixation in the mid-scapular region of the back was embedded by a polypropylene mesh (Evolution, BULEV, weight 48 g/mq, Dipromed, Italy). During the recovery period, at least 7 days after surgery, the catheters were flushed daily with 0.1 ml of gentamicin (40 mg/ml) and with heparinized saline (heparin 250 U/ml in 0.9% sterile saline).

Ten days after recovery from surgery, 13 rats were trained to SA 2-AG (25 μg/kg/20 μl, iv) in 1 h-daily sessions (5 days/week) for 13 consecutive sessions, according to a FR 1 schedule of reinforcement (FR 1, 1 nose poke: 1 injection). During the third week, when all rats had fulfilled the criterion of 85% responses in the active hole and stable responding over three sessions, the schedule of reinforcement was increased to FR 2 (2:1) (14th–24th session). A nose poke in the active hole resulted in a 4-s infusion of 2-AG. Each 2-AG infusion was followed by a 20-s time-out period, during which further nose pokes were recorded but did not result in additional intravenous infusions. Rats were also studied in the extinction phase from the 25th session, when the 2-AG solution was substituted with vehicle (25th–32nd day of administration). A group of six rats were also studied in reacquisition when vehicle was replaced with 2-AG (33th–40th). Another group of animals were used to study the effect of varying injection doses of 2-AG. At the end of each SA session, the catheters were flushed with 0.1 ml of heparinized saline. The responses performed by each rat on both holes for the entire 1-h daily session and the corresponding number of reinforces received was recorded (Graphic State 2 software, Coulbourn instruments, PA, USA).

### Microdialysis studies

Rats were anesthetized with Equitesin (3 ml/kg ip), prepared as previously described, and placed in a stereotaxic apparatus. The skull was exposed, and a small hole was drilled on one side. The probe was implanted vertically in the NAc shell (A + 2.2; L + 1.0 from bregma; V-7.8 from dura) or in the NAc core (A + 1.4; L + 1.6 from bregma; V-7.6 from dura) according Paxinos and Watson (55), and then fixed on the skull with dental cement. Rats were housed in transparent plastic (Plexiglas) hemispheric bowls with food and water available. Experiments were performed on freely moving rats 24 h after probe implantation. A Ringer’s solution (147 mM, NaCl; 2.2 mM, CaCl2; 4 mM, KCl) was pumped through the dialysis probe at a constant rate of 1 μl/min. Dialysate samples (10 μl) were taken every 10 min and injected without purification into an HPLC apparatus equipped with a reverse-phase column (C8 3.5 μm, Waters, Mildford, MA, USA) and a coulometric detector (ESA Coulochem II, Bedford, MA, USA) to quantify DA. The first electrode of the detector was set at + 130 mV (oxidation) and the second at −175 mV (reduction). The composition of the mobile phase was 50 mM NaH_2_PO_4_, 0.1 mM Na2-EDTA, 0.5 mM *n*-octyl sodium sulfate, and 15% (v/v) methanol, pH 5.5. The sensitivity of the assay for DA was 5 fmol/sample.

At the end of the experiment, animals were sacrificed and their brains were removed and stored in formalin (8%) before histological analysis. To this end, brains were cut on a vibratome in serial coronal slices (20 μm) oriented according to Paxinos and Watson (55) to locate the placement of the microdialysis probe.

### Data analysis

Nose pokes emitted during each 1-h 2-AG SA session during acquisition and extinction phases were analyzed by two-way repeated measures ANOVA with nose pokes (i.e., active vs inactive) and days as within subject factors. Reacquisition was analyzed by two-way ANOVA, with nose pokes (i.e., active vs inactive) and days with respect to the corresponding final 2-AG session (i.e., 32nd 2-AG SA session) as within subject factors. Where significant effects were obtained by ANOVA; LSD *post hoc* tests were performed.

Repeated measures ANOVA was applied to the data obtained from the serial assays of DA after each treatment. Results from treatments showing significant overall changes were subjected to *post hoc* Tukey tests with significance for *p* < 0.05. Basal values were the means of three consecutive samples differing less than 10%.

## Results

### Self-administration studies

#### Experiment 1: acquisition, extinction, and reacquisition of 2-AG self-administration

**Figure 1:**
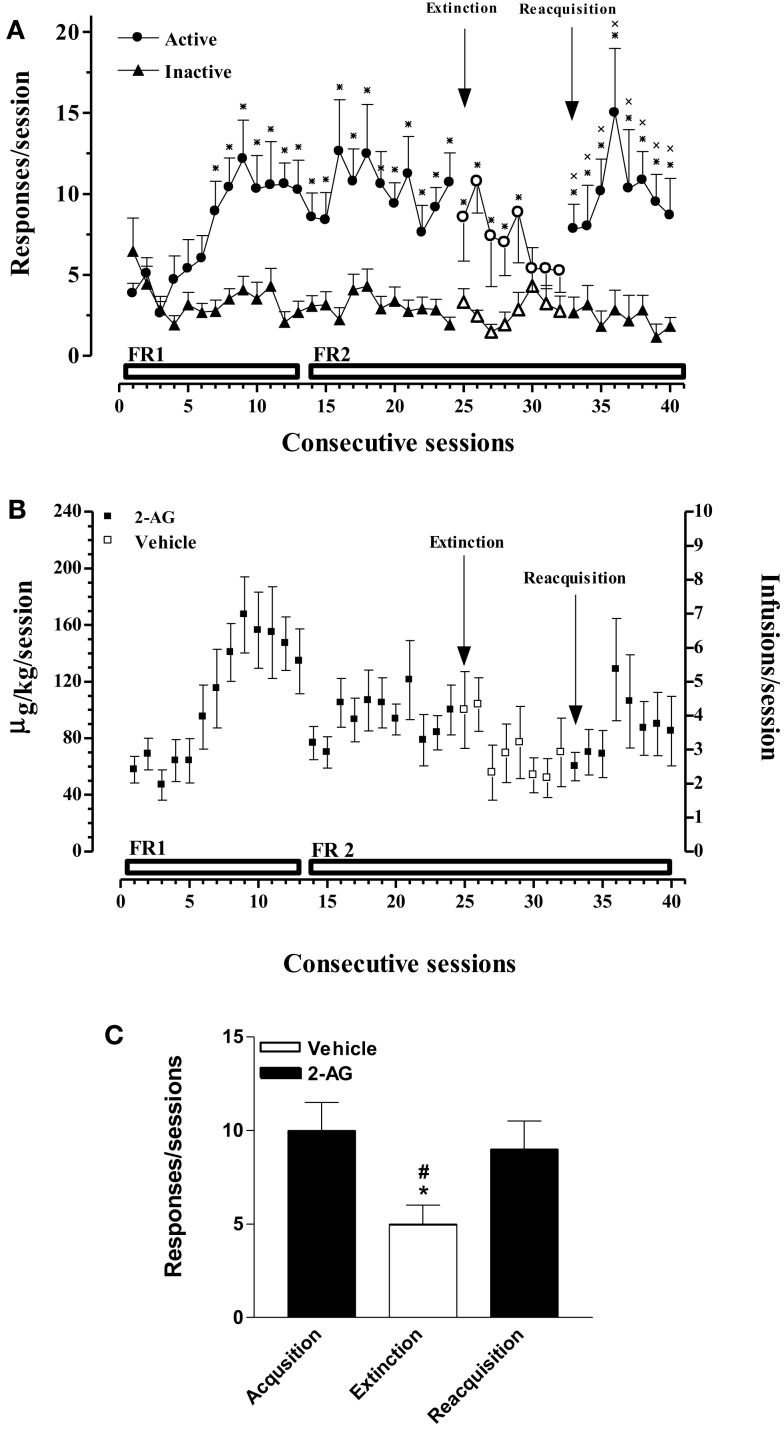
**Acquisition, extinction, and reacquisition of 2-AG self-administration (SA) behavior over consecutive session**. **(A)** Number of responses (nose pokes) for 2-AG SA (25 μg/kg/infusion). Results are expressed as mean ± SEM of nose pokes in the active (circle) and inactive (triangle) holes during each 1-h daily session under FR 1 and FR 2 schedule (acquisition phase: 1st–24th days, filled symbols, *N* = 13; extinction phase: 25st–32nd days, open symbols, *N* = 13; reacquisition phase: 33th–40th days, filled symbols; *N* = 6). *p < 0.05 vs inactive nose pokes; ANOVA followed by LSD *post hoc* test. **(B)** Daily intake and number of infusions during 2-AG SA. Data are expressed as μg/kg (left *Y*-axis) or number of infusions (right *Y*-axis) of 2-AG during each 1-h daily session (1st–24th; 33th–40th, filled squares) or vehicle (25th–32nd, open squares). **(C)** Number of responses during each phase of 2-AG SA. Each bar represents the mean ± SEM of the last three sessions under acquisition, extinction, and reacquisition phases of 2-AG SA. *p < 0.05 vs acq.; ^#^*p* < 0.05 vs reacq. ANOVA followed by LSD *post hoc* test.

In this experiment, acquisition, extinction, and reacquisition of 2-AG SA were studied. Figure 1A shows that rats implanted with a jugular catheter were trained to SA 2-AG (25 μg/kg/20 μl infusion, unit dose) in a single daily 1 h session, under an initial FR (FR) 1 schedule, which was then increased to FR2. Figure 1A also shows the average number of active and inactive nose pokes performed by rats trained on 2-AG SA during acquisition, extinction, and reacquisition phases. Two-way ANOVA of acquisition and extinction phases showed a significant effect of nose pokes (*F*_1,24_ = 3.466; *p* < 0.0001), and of sessions (*F*_31,744_ = 182.97; *p* < 0.0001) and a significant nose pokes × session interaction (*F*_31,744_ = 214.069; *p* < 0.0001). LSD *post hoc* tests showed significant differences between active vs inactive nose pokes from the 7th to the 29th 2-AG SA session. Two-way ANOVA of reacquisition, applied from the period 32nd to 40th session, showed a main effect of active vs passive nose pokes (*F*_1,10_ = 1381.47; *p* < 0.01). LSD *post hoc* tests showed significant differences between active and inactive nose pokes from the 33rd to the 40th 2-AG SA session. No differences were observed in active nose poking on each Monday following the weekend abstinence compared with the last session of the preceding week. The percentage of rats that acquired 2-AG SA was 90%.

Figure 1B shows the daily intake (μg/kg) of 2-AG or vehicle during all phases of SA (left *Y*-axis) and the corresponding number of reinforcements obtained (right *Y*-axis) in each 1-h session under a FR 1 or FR 2 schedule from rats that acquired 2-AG SA.

Figure 1C shows the mean number of responses during the last three sessions of acquisition, extinction, and reacquisition phases. One-way ANOVA showed a main effect of the different SA phases (*F*_2,15_ = 7.81; *p* < 0.05). LSD *post hoc* tests showed significant differences between extinction and acquisition or reacquisition.

#### Experiment 2: effect of dose of 2-AG and CB1 receptor blockade on 2-AG SA

**Figure 2:**
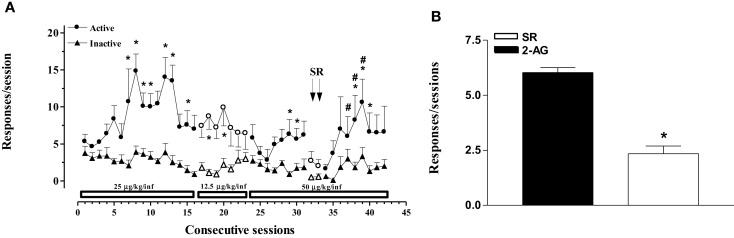
**2-Arachidonoylglycerol self-administration (SA) behavior at different 2-AG doses and effect of CB1 receptor blockade on SA**. **(A)** Number of responses (nose pokes) for 2-AG SA at varying 2-AG doses (12.5, 25, and 50 μg/kg) and effect of SR-141716A (1mg/kg, 30 min before sessions) on SA. Results are expressed as mean ± SEM of cumulative nose pokes in the active (circle) and inactive (triangle) nose pokes during each 1-h daily session of 2-AG SA: 25 μg/kg (1st–16th day); 12.5 μg/kg (17th–23rd day); and 50 μg/kg (24th–43rd day), *N* = 16. *p < 0.05 vs inactive nose pokes; ^#^*p* < 0.05 vs SR. ANOVA followed by LSD *post hoc* test. **(B)** Number of responses during 2-AG SA and effect of SR-141716A on SA. Bars represent the mean ± SEM of the last three sessions under 2-AG SA (50 μg/kg/inf) condition (black bar) and under SR pre-treatment condition for two consecutive session (SR, 1 mg/kg ip, 30 min before each session) (white bar). *p < 0.05 vs veh; ^#^*p* < 0.05 vs SR-50. ANOVA followed by LSD *post hoc* test.

In the second experiment, the effects of varying doses of 2-AG (12.5–50 μg/kg/20 μl infusion, unit dose) and rimonabant (SR-141716A, 1 mg/kg ip, 30 min before SA session) under an FR1 schedule were studied (Figures 2A,B). Two-way ANOVA of acquisition at the dose of 25 μg/kg/infusion showed a significant effect of nose pokes (*F*_1,24_ = 3.896; *p* < 0.0001) and of sessions (*F*_15,360_ = 324.115; *p* < 0.0001), and a significant nose poke × session interaction (*F*_15,360_ = 316.965; *p* < 0.0001). LSD *post hoc* tests showed significant differences between active and inactive nose pokes (7, 10, 12, 13, and 15th SA session). Two-way ANOVA of acquisition at the dose of 12.5 μg/kg/infusion showed a main effect of active vs passive nose pokes (*F*_1,15_ = 1.45, *p* < 0.001). LSD *post hoc* tests showed significant differences between active and inactive nose pokes (18, 20th 2-AG SA session). Two-way ANOVA of acquisition at the dose of 50 μg/kg/infusion showed a main effect of active vs passive nose pokes (*F*_1,15_ = 8.25; *p* < 0.01). LSD *post hoc* tests showed significant differences between active and inactive nose pokes (29, 30, 38–40th 2-AG SA session). Two-way ANOVA of acquisition at the dose of 50 μg/kg/infusion after rimonabant injection showed a significant main effect of active vs passive nose pokes (*F*_1,9_ = 6.22; *p* < 0.05) and of day (*F*_2,8_ = 2.19; *p* < 0.05). LSD *post hoc* tests showed significant differences between active and inactive nose pokes (37–40th 2-AG SA session).

Figure 2B shows the mean number of responses during the last three sessions of 2-AG SA at the dose of 50 μg/kg/infusion (29–31st 2-AG SA session) and the effect of rimonabant pre-treatment (SR, 1 mg/kg ip, 30 min before each session) for two consecutive sessions (32nd and 33rd 2-AG SA session). One-way ANOVA showed a main effect of the different SA phases (*F*_2,21_ = 9.91; *p* < 0.05). LSD *post hoc* tests showed significant differences between groups.

**Figure 3:**
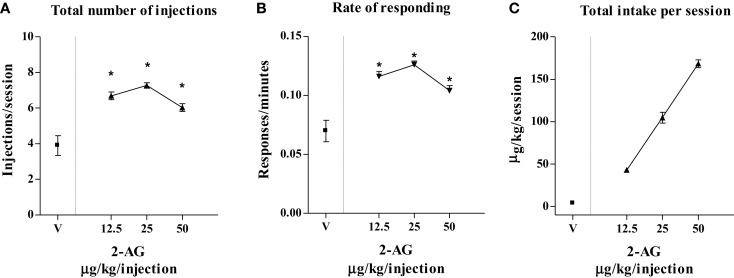
**Effect of varying injection dose on self-administration (SA) of 2-AG**. **(A)** Total number of injections of 2-AG (0, 12.5, 25, and 50 μg/kg/inf), **(B)** overall rate of responding for 2-AG, and **(C)** total 2-AG intake as shown as a function of injection dose of drug (0, 12.5, 25, and 50 μg/kg). Each point represents the mean ± SEM of the last three sessions under each 2-AG unit dose condition and under vehicle condition, *N* = 16. *p < 0.05 *post hoc* comparison vs veh (V).

Figure 3 shows that varying the injection dose of 2-AG resulted in a classic inverted-U-shape dose–response curve. One-way ANOVA showed that 2-AG maintained significantly higher numbers of infusions per session (*F*_3,8_ = 12.44; *p* < 0.01) (Figure 3A) and higher rates of responding (*F*_3,8_ = 45.75; *p* < 0.001) (Figure 3B) than vehicle at the doses of 12.5, 25, and 50 μg/kg/infusion. LSD *post hoc* tests showed that the maximal rate of responding (0.12 ± 0.09 response/s) and the number of injections per session (7 ± 1.19 injections/session) were maintained by 25 μg/kg 2-AG/infusion. The highest 2-AG intake per session was reached at a dose of 50 μg/kg/injection (155 ± 0.40 μg/session) (Figure 3C).

### Microdialysis studies

#### Effect of 2-AG administration on dopamine transmission in the NAc shell and core

Basal values of DA, expressed as fmoles/10 ml sample (mean ± SEM), were NAc shell 52 ± 5 (*N* = 19) and NAc core 54 ± 4 (*N* = 12).

**Figure 4:**
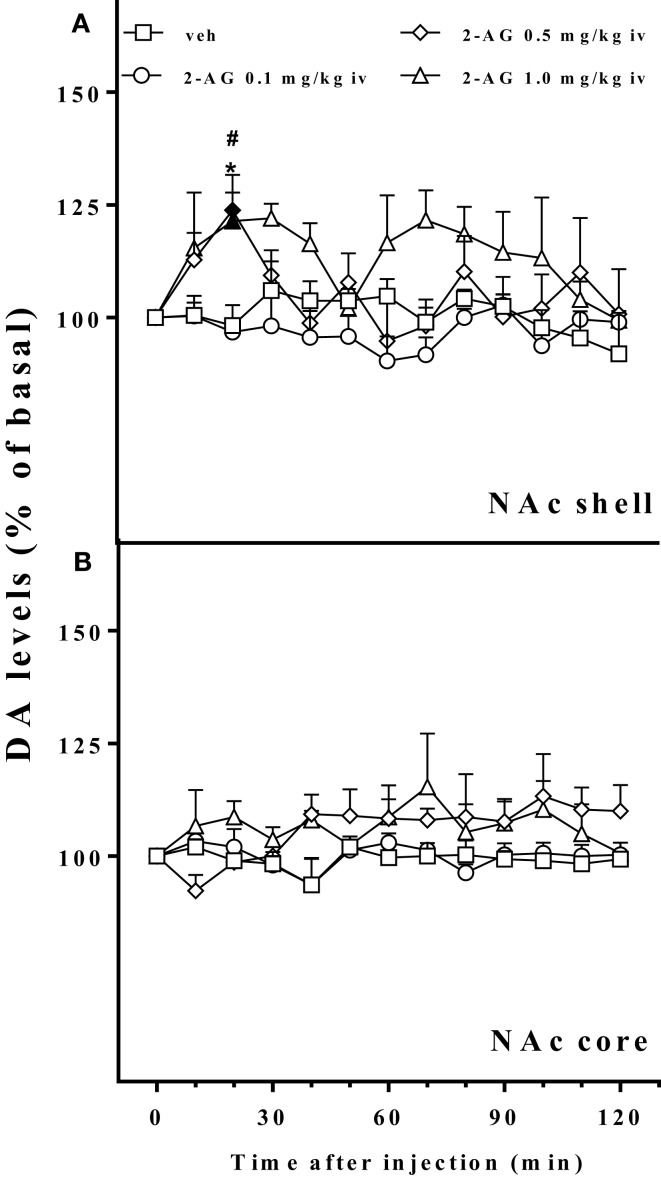
**Effect of 2-AG injection on nucleus accumbens dialysate dopamine**. Effect of 2-AG (0.1, 0.5, and 1 mg/kg iv) on NAc shell **(A)** and core **(B)** dialysate DA. Data are expressed as means (±SEM) of change in DA extracellular levels expressed as a percentage of basal values. Solid symbols: *p* < 0.05 with respect to basal values; *p < 0.05 vs veh group; ^#^*p* < 0.05 vs 0.1 group.

Figure 4 shows that 2-AG (0.1–1.0 mg/kg iv) preferentially stimulated the NAc shell with respect to the NAc core DA. The NAc shell DA increase was observed only at the higher doses tested (0.5 and 1.0 mg/kg iv) and was about 25% over basal value. One-way ANOVA of the 0.5 mg/kg dose showed a main effect of group (*F*_12,48_ = 3.27; *p* < 0.001). Tukey *post hoc* tests revealed differences at the 20 min sample with respect to basal value and to vehicle. One-way ANOVA of the 1.0 mg/kg dose showed a main effect of group (*F*_12,48_ = 2.17; *p* < 0.05). Tukey *post hoc* tests revealed differences at the 20 min sample with respect to vehicle and with respect to basal value. Three way ANOVA of DA release of the NAc shell and core implanted animals revealed a significant effect of dose (*F*_3,23_ = 4.9; *p* < 0.001) and a significant area × time interaction (*F*_12,276_ = 4.9; *p* < 0.05).

## Discussion

In the present study, we demonstrate for the first time that the eCB 2-AG is SA by Sprague-Dawley rats and stimulates DA transmission preferentially in the NAc shell as compared to the core. Hence, 2-AG possesses behavioral and DA releasing properties similar to those of food and drug rewards.

Initially, animals acquired 2-AG SA (25 μg/kg/20 μl infusion, unit dose) in single daily 1 h FR1 sessions for 5 weeks, which was then increased to FR2, under food restriction that maintained body weights at stable levels throughout these studies. Active nose pokes significantly increased over inactive nose pokes from the 6th SA session (acquisition phase), but no significant increase of nose pokes was observed under FR2. When 2-AG was replaced by vehicle (25th SA session, extinction phase), the responding rate as well as the number of injections slowly decreased. When the vehicle was replaced once again with 2-AG, SA behavior immediately recovered (reacquisition phase) (Figures 1A,C). In a second experiment, the effect of dose of 2-AG (12.5, 25, 50 μg/kg/20 μl infusion, unit dose) and the CB1 receptor-dependent SA behavior was assessed. Different doses of 2-AG showed a tendency toward a classic inverted-U-shape dose–response curve, although non-significant differences were observed between the 25 μg/kg dose and the other doses tested (Figure 3). The reinforcing effects of 2-AG in SA behavior were fully blocked by the administration of the CB1 receptor inverse agonist/antagonist SR-141716A (rimonabant, 1 mg/kg ip, 30 min before SA session) (Figure 2B). The average number of responses during each 2-AG SA session, however, was lower in the present study than the responses (either nose pokes or lever pressing) observed during SA studies using other cannabinoids or other common drugs of abuse. Thus, rats trained to SA WIN 55-212 (50, 56), nicotine (57, 58), cocaine (59, 60), or heroin (56, 61, 62) maintained higher rates of responding than what we observed for 2-AG SA. Nevertheless, our data are in agreement with a previous study showing that 2-AG is iv SA by non-human primates with either anandamide or nicotine SA history (40). Justinova et al. (40), however, observed that in squirrel monkeys the rate of responding maintained by 2-AG injections was similar to those observed in THC, anandamide, methanandamide, or cocaine SA studies (39, 63–65). In contrast with Justinova et al. (40), our experiments were carried out in naive animals. Thus, our findings indicate that 2-AG serves as an effective reinforcer and that its reinforcing properties are not dependent on previous SA of other drug reinforcers.

A second group of naive rats was used to estimate the neurochemical rewarding properties of 2-AG. Thus, 2-AG administered iv at 0.5 and 1.0 mg/kg doses was able to stimulate extracellular DA release preferentially in the NAc shell as compared to the core. The increase of DA in the NAc shell was about 25% over basal value, peaking at 20 min after the injection of 2-AG. DA stimulation in this area was absent after the lowest dose tested (0.1 mg/kg iv), and the DA levels were comparable to those after vehicle injections. Interestingly, the higher dose tested (1.0 mg/kg iv) elicited a biphasic DA response with a second rise in DA levels 60 min after 2-AG injection. Notably, our data show for the first time that 2-AG stimulates DA neurotransmission in the mesolimbic DA system. This feature is a common characteristic of all the drugs abused by human beings and it is believed to be crucial in the activation of brain rewarding pathways (66, 67), to be an anticipatory message for rewarding events (41) and to promote incentive learning (68) specially the DA increase in the NAc shell (42). This 2-AG-dependent increase of DA in the NAc shell most likely does not depend on the direct activation of DA neurons in the VTA, but, as in the case of THC and other cannabinoid agonists, is determined by the activation of CB1 receptors located on presynaptic glutamatergic and/or GABAergic terminals onto VTA DA neurons (69–71).

Previous studies have demonstrated that DA transmission increases in a dose-dependent fashion after administration of natural (THC), synthetic (WIN-55212), or endogenous (anandamide) cannabinoids and that this stimulation of DA in the NAc shell peaks at about 50% over basal levels. The doses used in these previous studies are comparable to those used in our study (49, 50, 52, 72) but they were accompanied by higher rates of SA behavior (39, 63, 64). In the present study, we did not observe a similar effect most likely due to the chemically unstable nature of 2-AG that quickly converts to 1-AG (38, 73). This, together with MAGL activity, could be responsible for the lack of high brain levels of 2-AG. Notably, in the present study, these properties of 2-AG were carefully taken into consideration during the preparation of the solutions for iv administration; 2AG content in administered solutions was determined daily before microdialysis or SA sessions by HPLC–MS/MS analysis.

Even though the rate of responding and DA stimulation were lower for 2-AG than those observed after administration of common drugs of abuse, our study demonstrates that the eCB system stimulation, via the mesolimbic DA system (microdialysis experiments) or by 2-AG itself (SA experiments), encodes rewarding events. The present microdialysis data, however, show that passive administration as a single intravenous bolus of 2-AG increased DA levels in the NAc shell at doses of 5–10-fold higher than the 2-AG intake reached by rats during SA behavior. A possible explanation might be the sensitization of the effect of 2-AG on DA transmission in the NAc shell of animals chronically exposed to 2-AG during the acquisition of 2-AG SA. Further experiments of SA coupled to microdialysis performed with the same experimental protocol previously published by our group (50) will serve to directly correlate the preferential increase of DA in the NAc shell to the 2-AG SA behavior observed in our experiments. On the other hand, the results obtained in 2-AG SA experiments may be also due to the food restriction regimen. Many studies show that the reduction of body weight by 20–25% dramatically enhances drug-induced SA (see (74) for review). Similarly, the suppression of natural growth used in the present study, although not associated with loss of weight, may have increased the motivation for 2-AG, suggesting that 2-AG SA may not be crucial in standard condition (e.g., *ad libitum* access to food). If so, the decrease of active responding for 2-AG after the administration of rimonabant could be also due to the appetite suppression induced by this drug (75).

Consistent with other studies (40, 52), the main significance of our findings is not that 2-AG may pose a risk of abuse liability, but that the activation of the eCB system together with the DA system may potentiate the reinforcing effects or the abuse potential of many drugs of abuse, supporting the hypothesis that an increase in eCB tone may be involved in drug-seeking behavior (76). Recent studies show that the manipulation of eCB levels in the brain can be selectively and successfully achieved by eCB clearance inhibitors (2, 77–79). Altogether, the above evidence from previous work, in conjunction with our current results, suggests that the eCB system plays a role in reward and reinforcement that makes it a good candidate as a therapeutic target in behavioral disturbances of motivation and reward.

## Conflict of Interest Statement

The authors declare that the research was conducted in the absence of any commercial or financial relationships that could be construed as a potential conflict of interest.

## Abbreviations

2-AG 2-arachidonoylglycerol; DA, dopamine; eCB, endocannabinoids; HPLC, high performance liquid chromatography; HPLC–MS/MS, high performance liquid chromatography–mass spectrometry; ip, intraperitoneally; iv, intravenously NAc, nucleus accumbens; SA, self-administration.
